# Images Enhancement of Ancient Mural Painting of Bey’s Palace Constantine, Algeria and Lacuna Extraction Using Mahalanobis Distance Classification Approach

**DOI:** 10.3390/s22176643

**Published:** 2022-09-02

**Authors:** Adel Nasri, Xianfeng Huang

**Affiliations:** State Key Laboratory of Information Engineering in Surveying, Mapping, and Remote Sensing, Wuhan University, Wuhan 430079, China

**Keywords:** mural paintings, area of paint layer loss, map deterioration, dark channel prior, mahalanobis distance, lacuna extraction, heritage institutions, documentation process

## Abstract

As a result of human activity and environmental changes, several types of damages may occur to ancient mural paintings; indeed, lacunae, which refer to the area of paint layer loss, are the most prevalent kind. The presence of lacuna is an essential sign of the progress of mural painting deterioration. Most studies have focused on detecting and removing cracks from old paintings. However, lacuna extraction has not received the necessary consideration and is not well-explored. Furthermore, most recent studies have focused on using deep learning for mural protection and restoration, but deep learning requires a large amount of data and computational resources which is not always available in heritage institutions. In this paper, we present an efficient method to automatically extract lacunae and map deterioration from RGB images of ancient mural paintings of Bey’s Palace in Algeria. Firstly, a preprocessing was applied using Dark Channel Prior (DCP) to enhance the quality and improve visibility of the murals. Secondly, a determination of the training sample and pixel’s grouping was assigned to their closest sample based on Mahalanobis Distance (MD) by calculating both the mean and variance of the classes in three bands (R, G, and B), in addition to the covariance matrix of all the classes to achieve lacuna extraction of the murals. Finally, the accuracy of extraction was calculated. The experimental results showed that the proposed method can achieve a conspicuously high accuracy of 94.33% in extracting lacunae from ancient mural paintings, thus supporting the work of a specialist in heritage institutions in terms of the time- and cost-consuming documentation process.

## 1. Introduction

**Figure 1:**
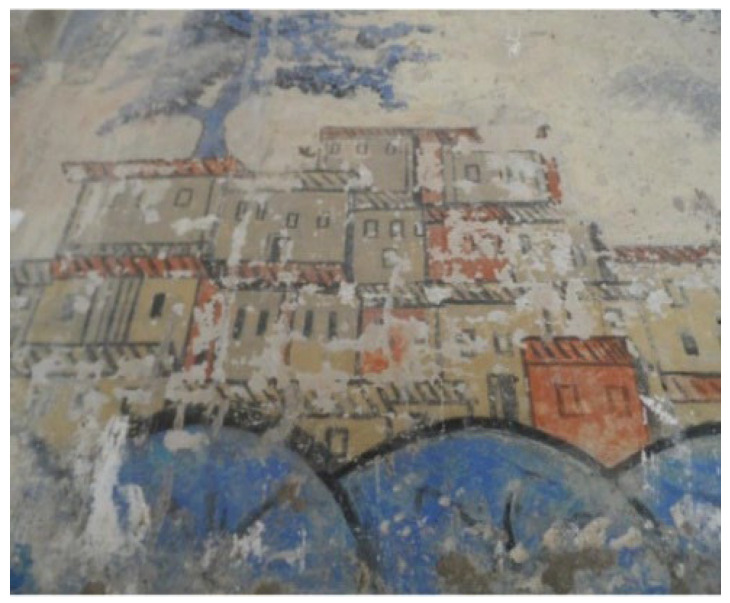
Examples of lacunae deterioration on the mural.

Ancient mural paintings [1] are known for their precious artistic and historic value. Algerian palaces are known for their rich content of mural paintings, such as the mural paintings of Bey’s Palace in Algeria. These paintings (covering about 2000 m^2^ of the surface) are an original reference document that states the story of a long journey of the master of the place, which can serve as a reference for researchers and historians. They certainly are the major attraction of the palace, showing their extraordinary character in Algeria and North Africa. They disclose many secrets that make it possible to read about many historical events such as the fights against the French army in which the Bey took part alongside the Dey of Algiers, in addition to the Bey’s many trips to the Middle East. These precious paintings are highly degraded and hard to maintain. Ancient mural paintings suffer from different degradation types due to natural impacts, changes in temperature and humidity [2], as well as human ignorance to preserve the paintings without knowing their value [3]. Most of the degradation are cracks, detachment, flacking, and so on. Lacunae are a typical type of damage caused by degradation. The lacunae regions, seen in Figure 1, can vary widely in size, from very small regions to large pores, and often have complex and irregular shapes.

Detection of lacunae areas is important for conservators in approximating the extent of the damage within the mural painting, which is required for documentation. For any conservation or restoration process of these mural painting the conservator or restorer must extract and map the degradation and find all the locations of damaged areas. Algerian Museums and Archaeology Departments map the deterioration manually. However, this is a time-consuming procedure, very tedious and low in accuracy [4]. Researchers are impatiently looking for ways to rapidly extract meaningful features with good accuracy. The progress of computation and image processing techniques provides great help for conservators and restorers in cultural institutions to extract the damaged regions in a non-destructive and automatic way. The potentiality of these processing tools led to the availability of diverse approaches for image processing. However, although the existence of new techniques such as deep learning can be very useful to extract unknown connections and detect hidden patterns. Deep learning requires a big amount of data and computational resources which are not always available in heritage institutions. The presence of many social and technological hurdles to the pollination between machine learning and cultural heritage becomes evident after reviewing the most recent literature concerning the relationship between the two disciplines [5]. The main part of these impediments has been created by some problems that are strongly associated with the quality and the access of datasets collected by researchers. These datasets are generally not large and not publicly available.

The main objective of this paper is to propose an efficient and robust approach to extract lacunae and map deterioration automatically from RGB images of ancient mural paintings of Bey’s Palace of Constantine in Algeria. As a first step, the RGB images of murals were preprocessed by using Dark Channel Prior (DCP), this step was needed to prepare the data for further processing and analysis. After preprocessing, the lacunae were extracted based on supervised classification of Mahalanobis Distance (MD).

The proposed approach confirms an efficient mural enhancement and lacunae extraction of ancient murals with the help of digital image processing techniques. We summarize the contributions of this paper as follows:

- In the literature, most studies have focused on detecting and removing cracks from old paintings. However, lacuna extraction has not received the necessary consideration and is not well-explored. Furthermore, most recent studies have focused on using deep learning for mural protection and restoration but deep learning requires a large amount of data and computational resources which is not always available in heritage institutions;
- The proposed Mahalanobis Distance classification approach is simple and robust. This approach does not require a large dataset, moreover it decreases the computational complexity and time-consuming manual classification and feature extraction of lacunae regions in ancient mural paintings of Bey’s Palace. Automatic extraction of large, degraded portions in ancient mural paintings of Bey’s Palace is a very challenging task. This is the first conducted study using image processing techniques to extract and map deterioration regions of Algerian mural paintings of various colors, sizes, and shapes in an automatic way, enhancing the quality and improving the visibility of the murals in terms of brightness and illumination by using Dark Channel Prior (DCP).

## 2. Related Works

In order to derive meaningful information from heritage data, scientists and conservation research institutions must conduct appropriate analyses and non-destructive methods [4,5]. This information will help scientists and conservation research institutions to identify new key research areas and optimize research activities [6]. In recent years, with the progress of machine learning approaches in image classification [7,8,9,10]. A wide range of new image acquisition methods [11]. Many tasks, such as painting style classification and forgery recognition [12,13], ancient coin classification [14], crack detection [15,16], cultural heritage collection indexing [17], missing color region extraction [18] have demonstrated the great potential of digital image processing in cultural heritage investigations. The common types of degradation that can be seen on ancient mural paintings are lacunae and cracks. Despite the progress of machine learning techniques applied in different applications, in the literature many approaches are utilized for the detection and removal of cracks in old mural paintings and lacuna extraction has not received much consideration. This section covers the numerous works that have focused on the detection and restoration of ancient mural paintings. Giakoumis et al. [15] developed an integrated technique for the restoration of cracks on a painting. The crack’s shape is characterized by the use of morphological top-hat transformation, while removing of the thin dusky brush strokes was performed by the use of semi-automatic technique of region growing or a median radial based neural network function. While for filling the crack they used anisotropic diffusion or order statistics filters. A new algorithm of lacuna region filling for virtual restoration of ancient Chinese paintings was proposed by Pei et al. [19] based on color contrast enhancement, they also used the Markov Random Field (MRF) model of texture synthesis. The technique adopts $u^{\prime }v^{\prime }$ the chromaticity domain of the L $u^{\prime }v^{\prime }$ color space, and the Y component of the XYZ color domain in addition to using adaptive histogram equalization to improve brightness in the Y component. Liu et al. [20] presented knowledge-based lacuna detection and segmentation for ancient paintings of Dunhuang murals in China. First, they trained a Bayesian classifier to execute a rough lacunae classification and control if there are lacunae in the provided mural images, then they segmented the lacuna areas by using the graph cuts image segmentation algorithm. Cornelis et al. [21] presented a fresh virtual method to detect and digitally remove cracks of the Ghent Altarpiece (1432) paintings. The crack’s detection is difficult due to variable content characteristics in different portions of the polyptych. Three innovative techniques are suggested and combined for detecting cracks of diverse dimensions, as well as for fluctuating brightness. The semi-supervised clustering method is used to clean and eliminate objects incorrectly labeled as cracks, while a patch-based technique is used to grip the noise of images and to raise the rendering to remove cracks for the subsequent painting phase. A new integrated system for digital restoration of wall paintings was implemented by Karianakis et al. [22]. The morphological feature location of the canny algorithm first finds the boundaries and identifies the missing parts. The following phase uses a Total Variation in-painting method. This method effectively restores only the small missing areas. A new method developed by Kaur et al. [16], is based on nearest neighbor for better extracting and eliminating the cracks. It is also possible to enhance the quality of wall painting pictures, increase and improve the quality of digital wall painting, and the white spots distortion is considered, which is extracted and cleaned. The near neighbor method is enhanced by raising hue as well as saturation. Compared with the SIHF system based on parameters that are peak signal to noise ratio (PSNR) and mean squared error (MSE), this system offers a more precise result and removes a greater number of cracks and white spots. Sinduja [23] presented a method of watermarking by detecting cracks in pictures of ancient sculptures and paintings of sanctuaries. Cracks are extracted by using thresholds, the result of the top-hat morphological transform. Then the thin, dusky strokes of the brush that were wrongly recognized as cracks are suppressed through the use of a semi-automatic method founded on the region growing. Cracks are categorized according to the unsupervised method, which includes fuzzy clustering. The K-means system can categorizes cracks on the exact class of cracks, and the watermarked information is stored. The user provides the crack category entry. The image’s output is the image of the watermark. The watermark information is extracted from the same crack category specified during the masking phase when collecting watermark data and the above crack identification method is performed.

An approach for mural deterioration detection using a multi-path convolutional neural network (CNN), was proposed by Huang et al. [9]. This method uses images of a scene with many lightings as inputs, after that it creates a binary map that indicates deterioration areas. The authors used seven-paths CNN for basic feature extraction from lighted images, and one path is used for cross-feature fusion. Moreover, they built two realistic mural deterioration datasets of real-world mural deterioration and briquettes that simulate the cave deterioration. Paint loss detection in digital painting “Ghent Altarpiece” by [24] is a recent and important study. First, this study proposed a general multimodal object detection technique based on sparse representation, in which the spatial features of various imaging modalities are merged in the kernel feature space through a kernel function. Additionally, spatial context is used by applying a smoothing filter to the representation residuals. Furthermore, a majority voting approach is introduced, overcoming the inevitable problem of imperfect annotation. A deep learning-based MCSN mural classification model was designed by [25] to classify murals under the effect of complex environments such as lighting and mural resemblance. The results show that the model improves the efficiency and accuracy of mural classification and can provide a convenient and time-saving method for scholars devoted to the identification and classification of mural dynasties, thus improving the effectiveness of mural classification. Pre-trained AlexNet and VGGNet models were used by Kumar et al. [26] for extracting features and classifying murals to address the insufficiency of existing mural datasets. Since the murals contain a lot of background noise, the extracting feature directly affects the classification quality.

As outlined above, this research area presents many motivating difficult tasks. Overall, the field of cultural heritage information extraction research is characterized by several issues. The artwork needs to be digitized once, in a way that makes a variety of applications easier. This places high demands on the acquisition equipment and methods. In addition, the real character of this research domain is that each work of art is unique in its nature: materials, dimensions, size, and procedures for works produced in various periods and by various artists can change enormously. The examined papers above show there is a great number of image processing techniques used for crack detection and removal of digital painting. Few studies have focused on lacuna detection in ancient mural paintings. Deep learning approaches require at least 1000 training cases which is not always available. They also require a high computation resource. In the case of ancient mural paintings in Algerian Museums and Archaeology Departments, they manually map lacunae regions which is also time-consuming and very tedious. To solve and address these drawbacks, we propose a simple and robust automated method to extract lacunae from mural paintings of Bey’s Palace. The proposed method detects lacunae of different sizes and shapes efficiently.

## 3. Materials and Methods

### 3.1. The Case Study

**Figure 2:**
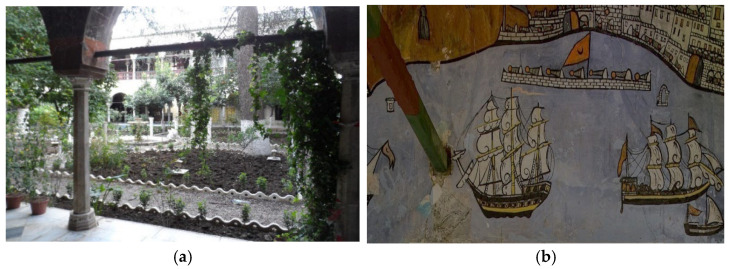
The Haj Ahmed palace: (**a**) the patio of Haj Ahmed palace; (**b**) the image depicting Ahmed’s pilgrimage to Mecca.

The Haj Ahmed palace, the Bey or sovereign of Constantine from 1826, illustrated in Figure 2, is one of the most beautiful buildings of the Ottoman period in Algeria. It has a succession of patios encircled by tiled arches and gardens of palm and orange trees, and ornamented with Tunisian and French tiles as well as mural paintings depicting Ahmed’s pilgrimage to Mecca. As shown in Figure 1, the mural paintings are in a very advanced state of degradation, the lacunae regions can be distinguished from the original colors by the naked eye.

### 3.2. Methods

**Figure 3:**
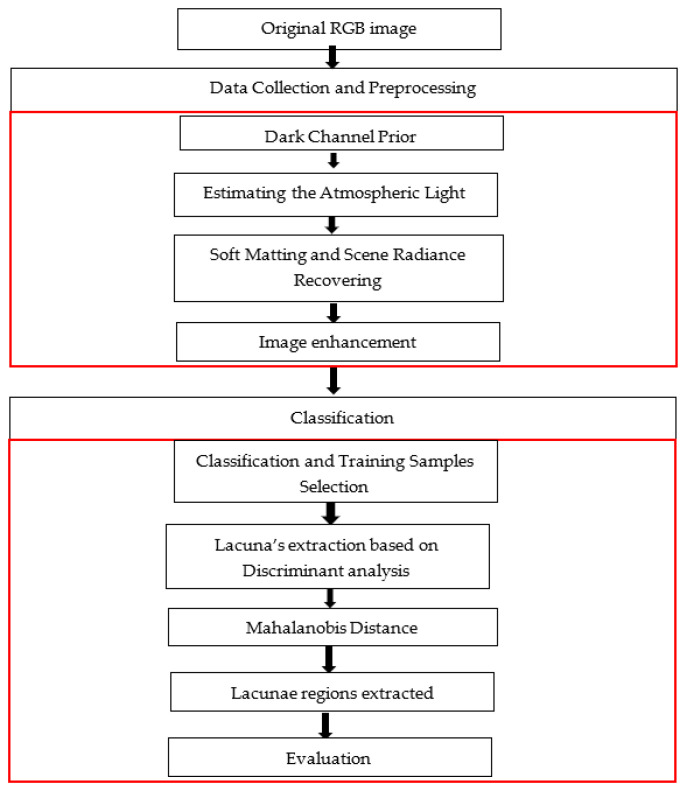
The overall workflow diagram of the proposed approach.

Figure 3 shows the overall workflow of the Mahalanobis Distance classification approach for the extraction of lacunae regions from ancient mural paintings of Bey’s Palace, including two main steps:

(1) Data collection and preprocessing using haze removal techniques [27] and Dark Channel Prior. Estimating the Atmospheric Light and the transmission, Soft Matting, and Scene Radiance Recovering;
(2) We started with the determination of the training sample by visual observation of the difference between the original colors of mural painting and the color of degraded areas as well as by manually examining the pixel intensity value of damaged regions. After Pixels grouping was performed by determining the closeness of each image pixel to each of the samples selected from the image, the pixels in RGB images were assigned to their closest sample based on Mahalanobis Distance. That measure was then used to determine the class of non-classified regions. Finally, we performed the evaluation of the obtained results. The details of each step are discussed in the following subsections.

### 3.3. Data Collection and Preprocessing

**Figure 4:**
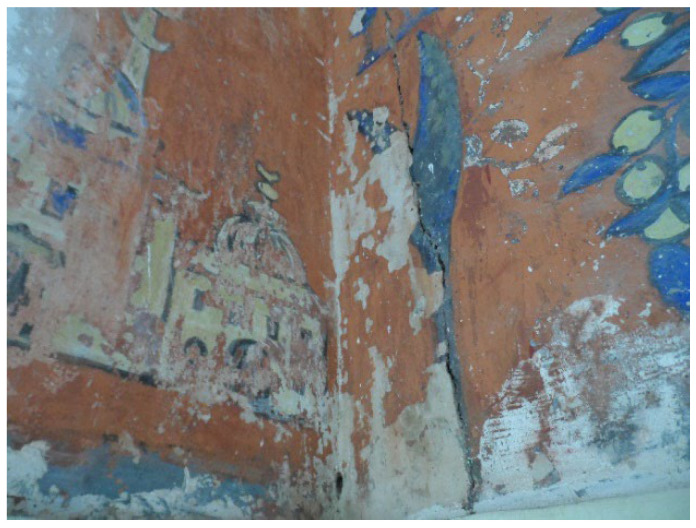
Un-enhanced image of mural painting of Haj Ahmed palace.

In this paper, six RGB images of the mural paintings were captured with different dimensions and characteristics. The RGB images of Ahmed Bey’s Palace were captured by using SAMSUNG camera model WB152F with a focal length of 4 mm. The images captured are severely affected and the colors are unbalanced as shown in Figure 4 due to low lighting circumstances. These images have poor dynamic ranges and excessive noise levels, which can harm computer vision algorithms’ overall performance. To make computer vision algorithms strong in low-light conditions, low-light image enhancement was used to improve the visibility of images. The histogram of pixel-wise inversion of low-light images or HDR images is very similar to the histogram of hazy images. Therefore, we can use haze removal techniques to enhance low-light images [28].

**Figure 5:**
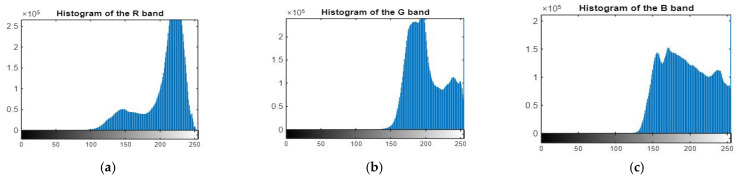
Histogram of R, G, and B bands of original image. (**a**) the R band, (**b**) the G band, (**c**) the B band.

By viewing the histogram of three bands, R, G, and B, as seen in Figure 5, we noticed that the data of the three bands are concentrated within a small part of the available dynamic range. This is one reason why the image appears hazy.

The main challenge related to our data is the unclearness of the content’s visibility. Typically, the visibility of this kind of data heritage is affected because it is captured under insufficient and unstable lighting conditions in the environment and due to unavoidable environmental problems such as the dust presented on murals as well as to some technical constraints during image capturing [29] where the conservator or restorer is unable to discern the true colors of the objects in these pictures [30]. The primary objective in this step is to overcome this problem by enhancing the quality and improving the visibility of the images which is needed for further processing and analysis.

### 3.4. Low-Light Image Enhancement

Image enhancement involves applying image processing techniques to adjust image properties, thus preparing the image for further analysis [31]. In this study we focused on using the method of haze removal similar to [32]. This method uses a Dark Channel Prior (DCP). The steps are: estimating the atmospheric light L using a Dark Channel Prior with estimating the transmission map T, refining the estimated transmission map, and restoring the image and performing optional contrast enhancement. The model to describe the haze of image I [33] is described as:

*I*(*x*) = *J*(*x*)*t*(*x*) + *A*(1 − *t*(*x*)) (1)

where *I*(*x*) is the hazy image, *J*(*x*) is the scene radiance of a haze-free image at *x*, *A* is atmospheric light, and *t*(*x*) is the medium transmission describing the portion’s light that reaches the camera.

#### 3.4.1. Dark Channel Prior

The DCP states that there is at least one pixel has very low intensity (dark pixel), where some pixel values are too close to zero. The DCP is defined as follows:

(2) $$J^{dark}\;(x)=\underset{y\in Ω\left(x\right)}{\min}(\;\underset{c\in \left(r,g,b\right)}{\min}J^{c}\left(y\right)),$$

where $J^{c}$ is denotes an intensity for a color channel *c* ∈ {*r*, *g*, *b*} of the three bands of RGB image and Ω(*x*) represents *x* patch locally centralized. For each patch in the image, centered at pixel *x*, the dark channel at *x* is defined as the lowest value for all the bands and all the pixels in that patch. The value of *J^dark^* should be low or very close to zero.

$$J^{dark}\to 0$$

#### 3.4.2. Estimating the Atmospheric Light and the Transmission

We calculated the average of the pixels in the original image that match to the top lightest one percent in the dark channel in order to obtain the atmospheric light. Hence, *A* has three elements, the average values for each color band. After we defined the transmission value of 1 in a local patch as constant and Ω the dark channel of the normalized haze image, we calculated the normalized haze image as the channel-by-channel division of each element by the matching channel of *A*.

#### 3.4.3. Soft Matting and Scene Radiance Recovering

The refined map can be calculated as follows: by using a Matting Laplacian matrix and solving sparse linear system [32]:

(3) $$\left(L+\lambda U\right)t=\lambda \bar{t}$$

where λ is a constant, *t* is the refined transmission and $\bar{t}$ the transmission of the original image. Finally, after obtaining the transmission map *t* with the atmospheric light *A*, Equation (4) can be used to recover the scene radiance *J* [32].

(4) $$J(x)=\frac{I\left(x\right)-A}{\max\left(t\left(x\right),\;t_{0}\right)}+A$$

where we set $t_{0}$ as a constant value to avoid dividing by 0.

### 3.5. Classification and Training Samples Selection

The image classifications techniques were used by remote sensing specialists following certain approaches such as supervised and unsupervised techniques. Supervised classification needs precise human intervention for feature extraction [34]. In supervised classification, the user identifies classes, then offers training samples of each class for the machine learning algorithm to use when classifying the image. This method works well if the user has a good understanding of what classes are present in their region of interest or he is looking for the presence of specific classes. Unsupervised classification does not use training samples or a provided set of classes. Image classification is a powerful algorithm that uses machine learning to train samples to identify the classes of lacunae in the entire image. After image enhancement, the determination of the training sample was carried out in situ by visual observation of the difference between original colors of mural painting and color of degraded areas as well as by manually examining the pixel intensity values of damaged regions seen in these precious murals. The mural is a 3D array of RGB images (a combination of red, green, and blue). Each color has its value between 0 and 255. The color can be found by combining the values in each of the three layers.

**Figure 6:**
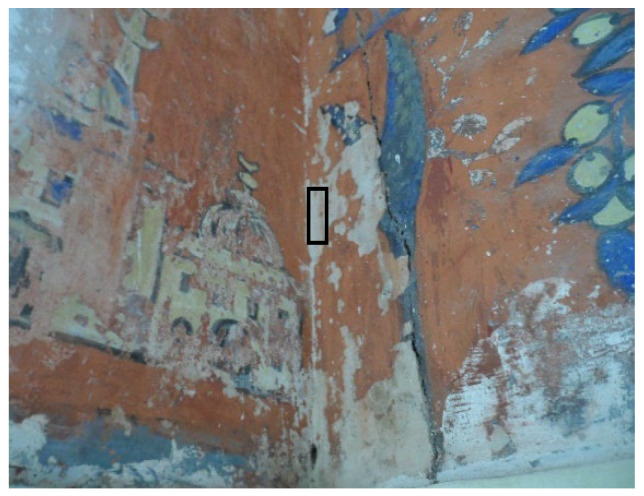
ROI (in rectangle) in the original RGB image.

Based on our classification schema for lacunae extraction from our data, two classes are defined. It is supposed that the majority of pixels belonging to lacunae are on the missing color regions (ROI), represent class 1 and all the remaining pixels belonging to non-lacunae are on the original colors grouped in class 2. Figure 6 shows an example of ROI enclosed in a black rectangle. The training sample was then used as input model for extracting the region of interest.

#### 3.5.1. Lacuna’s Extraction Based on Discriminant Analysis

The discriminant analysis method is used for supervised statistical classification. Originally developed for multivariate normal distribution data, it is utilized in machine learning as well as other applications that deal with pattern recognition. After having defined the classes based on their color information, the other pixels outside the training regions must be assigned to these classes. So, these training samples are references for the classification of all other pixels in the entire image. The approach was used to assign left pixels to the right class named the Mahalanobis Distance (MD) classification.

#### 3.5.2. Mahalanobis Distance

**Figure 7:**
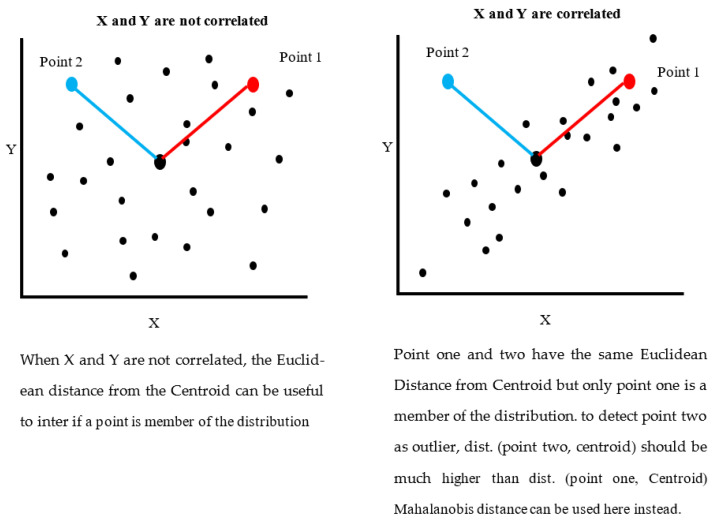
The difference between Euclidean and Mahalanobis Distance and how the Mahalanobis Distance is calculated using the variances of data points in RGB space.

The Euclidean distance is an ordinary distance between any two points, and is a popular method for measuring color distances [35]. Because the Euclidean distance takes red, green, and blue bands into account on the same scale, it may not be the best measurement for color distances. As a result, Mahalanobis Distance is preferred over Euclidian distance as it is shown in Figure 7, since it considers point distribution (correlations).

The Mahalanobis Distance classification is a direction-sensitive distance classifier that uses statistics for each class and was introduced by P.C. Mahalanobis in 1936 [36]. Moreover, Mahalanobis Distances are based on calculation of both the mean and variance of the classes in three bands (R, G, and B), and is the covariance matrix of all the classes; therefore, it takes into consideration the fact that the variances in each direction differ, furthermore it takes into consideration the covariance among variables. Note that the variance in each direction of each channel R, G, and B, was different. For this reason, a classifier that considers such differences is needed, such as Mahalanobis which was used in this study.

As we mentioned above, samples were selected based on the number of classes in the image and the samples data were used to determine a class model for each mural to perform supervised classification. After the pixels grouping process is performed by determining the closeness of each image pixel to each of the samples selected from the image, the pixels in RGB images are assigned to their closest sample based on Mahalanobis Distance. The Mahalanobis Distance classification is in the equation used in [37] to compute the distance between each pixel in the image and each class.

(5) $$d\left(x,y\right)=\sqrt{{\left(x-y\right)}^{t}C^{-1}\left(x-y\right)}$$

where *d* is Mahalanobis Distance, *x* is a data point in the 3-D RGB space, *y* is the mean vector of a sample in an RGB image, and $C^{-1}$ denotes the inverse of the covariance matrix of the sample.

The Mahalanobis color distance normalizes the impact of each feature’s distribution by taking into account the correlation between each pair of terms [36].

For RGB color images *C* is computed as:

(6) $$C=\left[\begin{array}{ccc} \sigma_{RR} & \sigma_{RG} & \sigma_{RB}\\ \sigma_{GR} & \sigma_{GG} & \sigma_{GB}\\ \sigma_{BR} & \sigma_{BG} & \sigma_{BB} \end{array}\right]$$

And the element *C* then can be calculated as follows:

(7) $$\sigma_{RG}=\sigma_{GR}=\sigma_{BR}=\frac{1}{n-1}\sum_{i=0}^{n}\;(R_{i}-\bar{R}\;)\left(G_{i}-\bar{G}\right)$$

The same formula can be used with channel *R* and *B* and channel *G* and *B*.

Where $R_{i}$, $G_{i}$, and $B_{i}$ are the values of the *i*th match (*I* = 1, 2, 3, 4, …, *n*); $\bar{R}$, $\bar{G}$, and $\bar{B}$ are the mean color values for *R*, *G*, and *B* in the provided image, respectively.

The effect of *C* was to scale the distance along each feature, in our case each channel *R*, *G*, and *B* was considered as a feature.

### 3.6. Accuracy Evaluation

The accuracy assessments afford the majority of information on where the errors of classification occurred. To know the accuracy of a classification and evaluate the effectiveness of our proposed approach, the method was applied to each of the six murals to generate a range of lacunae extraction images; this algorithm classifies images and then evaluates pixel by pixel according to the dividing sum of the number of pixels extracted by the total number of registered pixels in order to calculate the misclassification rate which is defined as follows:

(8) $$\mathrm{Misclassification}\;\mathrm{rate}\;\%=\frac{\mathrm{number}\;\mathrm{of}\;\mathrm{pixels}\;\mathrm{extracted}\;}{\mathrm{total}\;\mathrm{number}\;\mathrm{of}\;\mathrm{registered}\;\mathrm{pixels}}\times 100$$

## 4. Results and Discussion

### 4.1. Image Enhancement

MATLAB 2021b version 9.11 was used to carry out the implementation and processing of all the mentioned steps.

**Figure 8:**
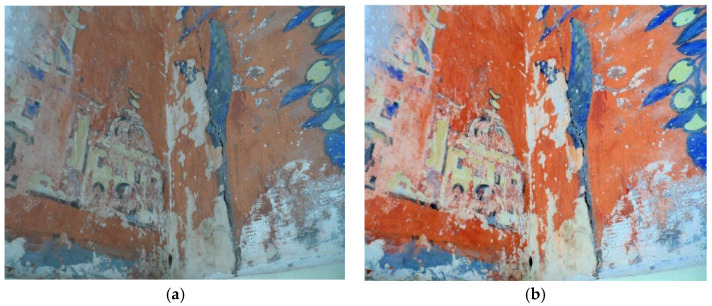
Display result of the dehazing method applied on mural painting of Haj Ahmed palace side-by-side, (**a**) input haze image, (**b**) output image.

**Figure 9:**
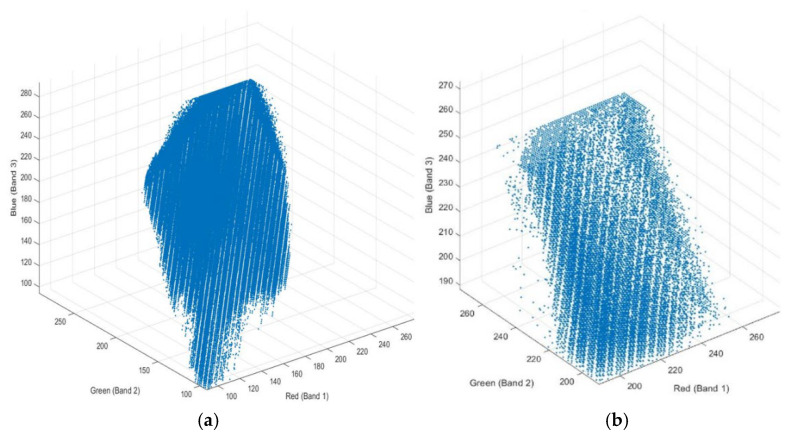
The 3D plot of R, G and B bands, (**a**) un-enhanced image, (**b**) image enhanced.

**Figure 10:**
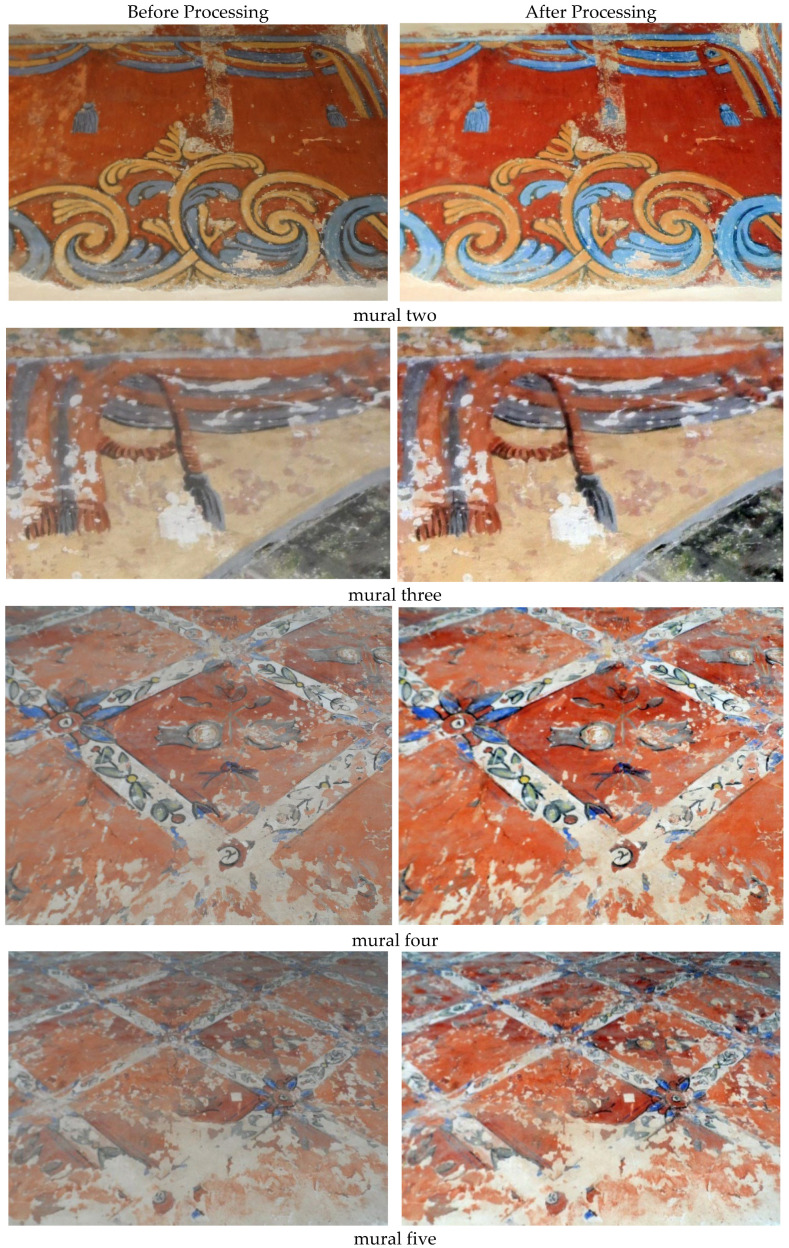

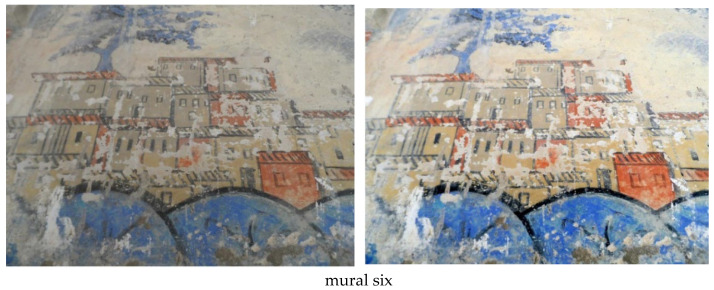
The original and the results of enhancing of murals 2, 3, 4, 5, and 6 using DCP.

It is difficult to clearly distinguish between colors which affected visibility in the haze of the original image, as seen in Figure 8a. Another reason for the hazed appearance of the image is that the bands are highly correlated with each other. The three-band scatterplots are an excellent way to measure the degree of correlation among the bands. It can be seen from the image Figure 8b that the algorithm is very useful in image enhancement. Figure 8 shows that the hazy effect was removed, and the image quality, colors, and details was enhanced which improved its visibility and clarity in terms of brightness and illumination. Figure 9 shows the scatterplot of R, G, and B bands of enhanced and un-enhanced mural one. In Figure 10 we present a similar result for the rest of the murals enhancement.

### 4.2. Lacuna Extraction Based on Mahalanobis Distance

The extraction of lacuna regions from ancient mural paintings is mainly challenging due to their huge variation in size, shape, and intensity as well as their complicated background. Our proposed MD approach was applied to six color images corresponding to various sizes and characteristics. The set of images was processed using Mahalanobis Distance to assess pixel color similarities to extract our region of interest.

**Figure 11:**
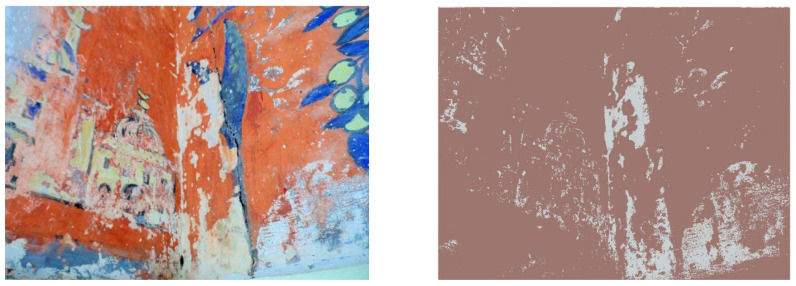
The original and the output of mural one. The MD approach successfully extracted the lacuna regions.

**Figure 12:**
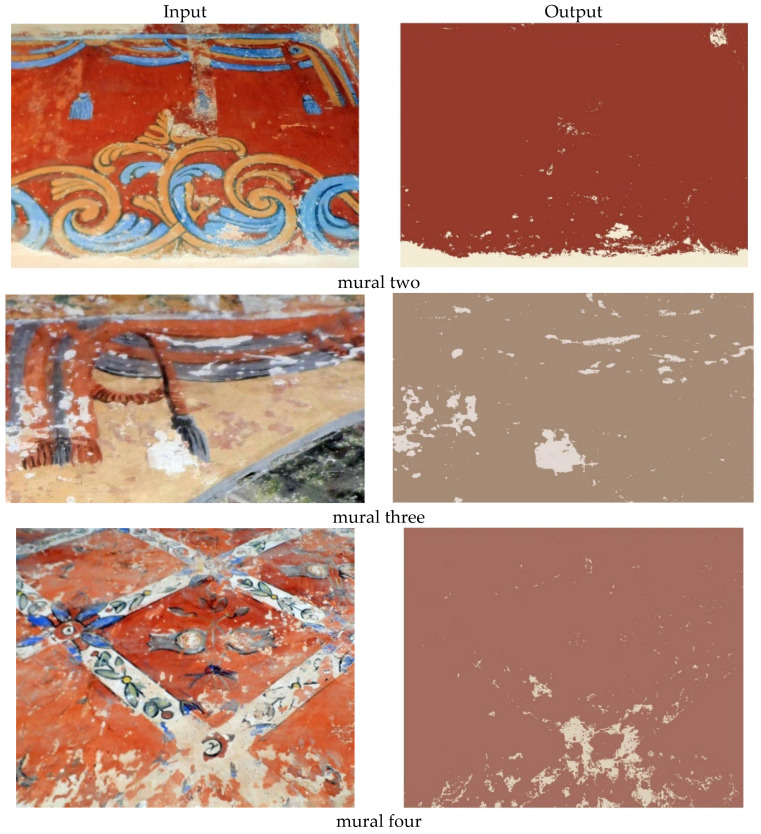

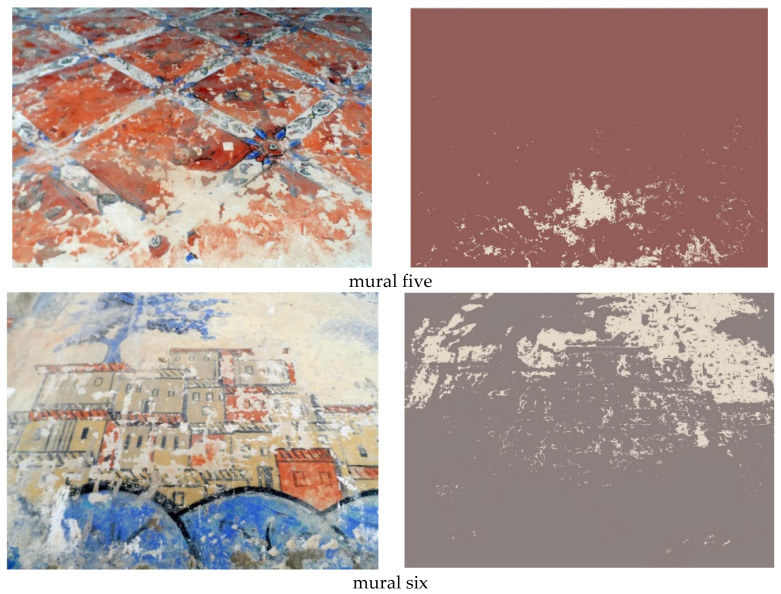
The original and the output of murals 2, 3, 4, 5, and 6. The MD approach successfully extracted the lacunae regions.

By using the average mean of each color channel in RGB space, we take into account the different characteristic colors of each image. For each class (each element presents in the images), RGB values of 4112 pixels were registered as a reference for lacuna class and 86,240 pixels were registered as a reference for non-lacunae class. For the image classification, each pixel of the images was assigned to the most proximate class according to Mahalanobis Distance. This distance takes into account the covariance between variables (red, green, and blue (RGB) values) which was reported to induce misclassifications on RGB images. Figure 11 shows the output for mural one of lacunae extraction through Mahalanobis Distance. According to the results shown in Figure 11, lacunae regions of mural one were extracted successfully by using Mahalanobis Distance. In Figure 12, we present a similar result for the rest of the murals extraction.

Table 1 illustrates the number of registered pixels for each class (lacunae class and non-lacunae class) in addition to the calculated of the mean values of each color channel (red, green, and blue).

The rest of the results are shown in the following Figure 12.

Using visual assessment in Figure 11, the lacunae regions were successfully extracted with a relatively lower number of misclassified pixels. This result also showed that the proposed method can be used in the extraction of lacunae from ancient mural paintings.

As we mentioned above, the result in Figure 12 shows that the presented lacuna extraction method has a good performance in discriminating the lacuna regions from other regions and keeping the space coherent of lacuna regions. Table 2 shows the classification summary and the number of extracted pixels after applying the MD approach.

As shown in Table 2, we calculated the number of extracted pixels for each class; a common difficulty in extracting lacuna regions are the pixels missed for each class extraction. However, the accuracy of 94.33% is high.

Our region of interest is the lacunae class, the extracted pixels are shaded in grey for each other class of the image. The misclassification rate was calculated for each image. The obtained result of mural one through Mahalanobis Distance is provided as follows: the misclassification rate accounted for 2.12%, by dividing the sum of the number of pixels extracted 8717 by the total number of registered pixels 4112, which provided an overall accuracy of 97% calculated by subtracting the result of the misclassification rate from one hundred percent.

Several factors have a significant impact on extracting the paint loss areas and must be taken into account in the estimation of these loss areas. We note the given complicated background, some uneven illumination impact and the number of training samples taken for each class as major factors influencing the extraction of the paint loss areas.

The findings reveal a significant degree of diversity in accuracy over a wide range of images. Murals one and two, in particular, had extremely high accuracy of 98%, this is hardly surprising as these images exhibited relatively clear identifiable lacuna regions from non-lacuna with minimal background data resulting in accurate extraction.

Murals four, and five proved difficult while using the proposed approach in lacuna extraction. These murals displayed a consistent accuracy of approximately 92% due to the pixel values of the white color of some regions of background from the non-lacunae class having very close pixel values to the lacunae class.

Some similar difficulties were experienced with murals three and six which showed the lowest extraction accuracy of 92% and 90%, respectively. This mural included a complex background in which some regions are of a similar color to those of lacunae regions and were therefore difficult to extract reliably.

#### Method Evaluation on Ancient Mural Paintings of Mogao Caves

We also evaluated our proposed approach on various images of ancient mural paintings of Mogao Caves in China. These data were provided by Wuhan Dashi Smart Technology company [38]. In the 20th century, Dunhuang Mogao Caves were considered one of the greatest cultural discoveries of all time because of their colorful mural paintings. The effects of time, physics, and human activity lead to generate lacunae and many kinds of deterioration to these priceless mural paintings. The data provided by Daspatial are high-resolution RGB images that were not preprocessed of four murals of complex backgrounds with different spatial size as follows: mural one with a dimension of 2911× 3799, mural two with a dimension of 2779 × 3313, mural three with a dimension of 2929 × 1991, and mural four with a dimension of 2505 × 1880.

**Figure 13:**
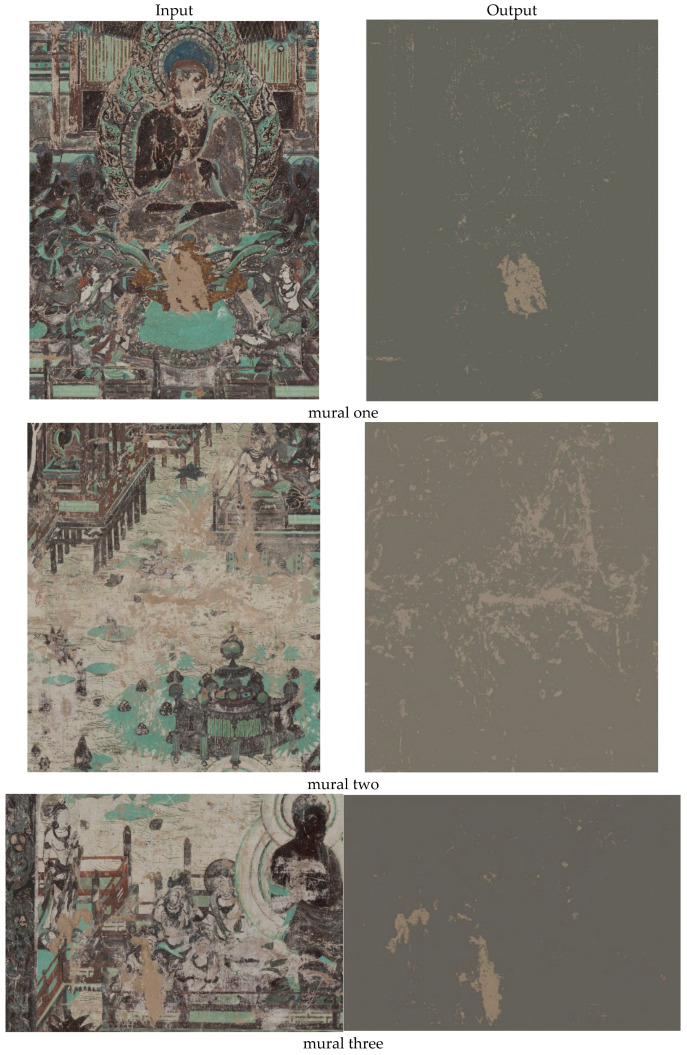

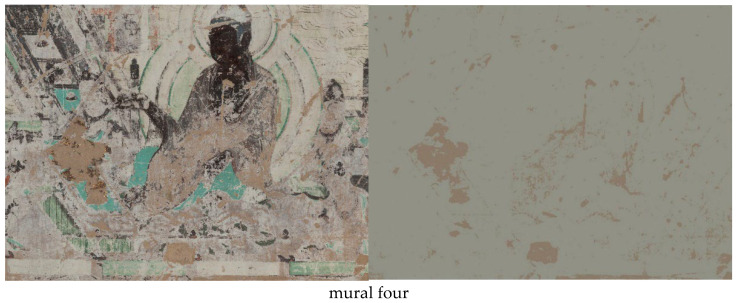
The four mural paintings of Mogao Caves and their outputs.

The visual assessment clearly shows the effectiveness of the proposed method. The results are presented in Figure 13.

Table 3 confirms that our proposed approach achieves a high extraction performance of 93%.

Compared with the results extraction of mural paintings of Bey’s Palace in Algeria, Mogao Caves has nearly the same extraction performance.

## 5. Conclusions

In this paper, we addressed the challenging problem of lacunae extraction and map deterioration in ancient mural paintings of Bey’s Palace in Algeria and mural paintings of Mogao Caves. We first preprocessed the RGB images of murals by using Dark Channel Prior (DCP) because they were captured under insufficient and unstable lighting conditions as well as in an unavoidable environment. So, the primary objective of this step was overcoming this issue by enhancing the quality and improving the visibility of the murals. Then, we determined two classes: lacunae and non-lacunae with training samples for each class. These training samples were references for the classification of all other pixels outside the training regions for the entire image. We used Mahalanobis Distance (MD) classification to assign left pixels to the right class by calculating the mean and variance of the classes in three channels (R, G, and B) and the covariance matrix of all classes. Experimental results on images captured of Bey’s Palace reveal the efficiency of the proposed approach, where the classification performance for the extraction of lacunae regions was very high and their effectiveness exceeded 94%. This approach shows its potential to support heritage institutions in the conservation and digital documentation process.

Many factors have a significant impact on extracting the area of paint loss such as: complicated background, some uneven illumination, and surface conditions of the murals as well as the number of training samples taken for each class. All these factors are considered open issues in the literature for extracting the paint loss areas. As perspectives, we have considerations to extend this work in depth and face the limitations of the proposed approach such as the extraction of loss regions of depth layers. In addition, special care of selecting samples must be considered, this will inevitably lead to less confusion during the classification process. We also plan to investigate other types of classification such as minimum distance and maximum likelihood or context-based methods.

## Figures and Tables

**Table 1:** Number of registered pixels for each class and the calculated of the mean values of each color channel (red, green, and blue).

Image	Classes	Number of Registered Pixels	Red Mean	Green Mean	Blue Mean
Mural one	Class 01	86,240	158.31	118.02	112.58
	Class 02	4112	201.16	208.94	209.24
Mural two	Class 01	124,788	150.30	59.81	44.08
	Class 02	28,002	245.58	236.36	214.84
Mural three	Class 01	115,604	166.97	135.04	113.84
	Class 02	4358	226.96	214.30	205.86
Mural four	Class 01	55,869	165.42	108.41	96.95
	Class 02	4937	223.17	211.20	189.72
Mural five	Class 01	26,538	146.69	94.61	87.84
	Class 02	3479	229.46	221.52	202.06
Mural six	Class 01	65,723	141.31	129.68	127.86
	Class 02	2730	228.75	219.51	203.16

**Table 2:** Classification summary and the number of extracted pixels after applying the MD approach.

Image	Classes	Number of Registered Pixels	Total Number of Registered Pixels	Number of Extracted Pixels	% Correct	Misclassification Rate (% Error)
Mural one	Class 01	86,240		182,828		
	Class 02	4112	90,352	8717	97.88	2.12
Mural two	Class 01	124,788		41,180	99.67	0.33
	Class 02	28,002	152,790	9240		
Mural three	Class 01	115,604		895,931		
	Class 02	4358	119,962	33,774	92.25	7.75
Mural four	Class 01	55,869		507,849		
	Class 02	4937	60,806	44,877	90.91	9.09
Mural five	Class 01	26,538		148,082		
	Class 02	3479	30,017	19,412	94.42	5.58
Mural six	Class 01	65,723	68,453	599,393	90.88	9.12
	Class 02	2730		24,897		

**Table 3:** Classification summary and the number of extracted pixels after applying the MD approach of mural paintings of Mogao Caves.

Image	Classes	Number of Registered Pixels	Total Number of Registered Pixels	Number of Extracted Pixels	% Correct	Misclassification Rate (% Error)
Mural one	Class 01	787,347	818,038	4,007,596	94.91	5.09
	Class 02	30,691		156,217		
Mural two	Class 01	639,917	654,041	10,353,857	83.82	16.18
	Class 02	14,124		228,526		
Mural three	Class 01	318,799	342,280	321,986	98.99	1.01
	Class 02	23,481		23,715		
Mural four	Class 01	314,031	333,978	813,340	97.41	2.59
	Class 02	19,947		51,662		

## Data Availability

The datasets generated during and/or analyzed during the current study are available from the corresponding author on reasonable request.
